# Access to University Mental Health Services: Understanding the Student Experience: L’accès aux services universitaires de santé mentale : comprendre l’expérience des étudiants

**DOI:** 10.1177/07067437241295640

**Published:** 2024-11-04

**Authors:** Nathan King, William Pickett, Kurtis Pankow, Gina Dimitropoulos, Emma Cullen, Stephen McNevin, Scott B. Patten, Anne Duffy

**Affiliations:** 1 Department of Psychiatry, 4257 Queen's University, Kingston, Ontario, Canada; 2 Department of Public Health Sciences, 4257 Queen's University, Kingston, Ontario, Canada; 3 Department of Health Sciences, 7497 Brock University, St. Catharines, Ontario, Canada; 4 Department of Sport and Exercise Science, 7759 Swansea University, Swansea, Wales, UK; 5 Department of Psychiatry, Mathison Centre for Mental Health Research & Education, 2129 University of Calgary, Calgary, Alberta, Canada; 6 Faculty of Social Work, 2129 University of Calgary, Calgary, Alberta, Canada; 7 Department of Psychiatry, 6396 University of Oxford, Oxford, UK

**Keywords:** university, mental health services, student, student health services, access to care, barriers, gaps, student experience, université, services de santé mentale, étudiant, services de santé étudiants, accès aux soins, barrières, lacunes, expérience étudiante

## Abstract

**Objective:**

To describe student access to university mental health services and barriers and gaps in support.

**Methods:**

This multiple cohort study used self-report data from 4,138 undergraduate students who completed the U-Flourish Well-Being Survey at the start and completion of first year from 2018 to 2023. The survey incorporated validated measures of mental health symptoms, barriers to care, and open-text questions about the mental health care experience and perceived gaps. Quantitative analyses summarized utilization patterns and barriers. An interpretive qualitative analysis identified common themes about support services and opportunities for improvement from the student perspective.

**Results:**

At university entry, 43% of students screened positive for anxiety and/or depression, 30% reported a lifetime mental disorder and 23% a lifetime history of self-harm. Over first year, 15% of students surveyed accessed university mental health services. Access was more likely in students identifying as older, gender diverse, female, having a prior mental disorder and those who screened positive for anxiety or depression. Common attitudinal and practical barriers reported included *thinking problems would resolve* (74%), *being uncomfortable sharing* (73%), and *not knowing how to get help* (50%). Common stigma barriers included *concerns about what family or friends might think*. Students expressed that both campus-based well-being and mental health care offered during flexible hours and accessible through online booking were important.

**Conclusions:**

Student-tailored mental health literacy may be a sustainable approach to address the attitudinal and practical barriers identified. If such barriers are reduced, an increased service demand would be expected and improved efficiencies needed. A clear Statement of Services, an online singular point of access with embedded triage to signpost students to indicated levels of care, and clearly worked-out care pathways including to community-based services would better align with a stepped care model, improve efficiency and access, and foster realistic expectations around university mental health support.

## Résumé

**Objectif:**

décrire l’accès des étudiants aux services universitaires de santé mentale, ainsi que les obstacles et les lacunes en matière de soutien.

**Méthodes:**

cette étude multi-cohortes a utilisé les données autodéclarées de 4,138 étudiants de premier cycle qui ont répondu au sondage U-Flourish Well-Being au début et à la fin de la première année de 2018 à 2023. Le sondage comprenait des mesures validées des symptômes liés à la santé mentale, des obstacles aux soins, ainsi que des questions ouvertes sur la façon dont les soins de santé mentale sont vécus et sur les lacunes perçues. Des analyses quantitatives ont résumé les schémas d’utilisation et les obstacles. Une analyse qualitative déductive a permis de dégager des thèmes communs sur les services de soutien et les possibilités d’amélioration du point de vue des étudiants.

**Résultats:**

à leur entrée à l’université, 43% des étudiants ont obtenu un résultat positif au dépistage de l’anxiété et/ou de la dépression, 30% ont déclaré avoir souffert de troubles mentaux au cours de leur vie et 23% ont dit s’être automutilés au cours de leur vie. Au cours de la première année, 15% des étudiants interrogés ont eu recours aux services de santé mentale de l’université. Les étudiants s’identifiant comme étant plus âgés, appartenant à la diversité de genre, de sexe féminin, ayant déjà souffert de troubles mentaux, et ceux qui ont obtenu un résultat positif au dépistage de l’anxiété ou de la dépression étaient plus susceptibles de recourir aux services. Les obstacles comportementaux et pratiques les plus courants étaient les suivants : *penser que les problèmes vont se résoudre d’eux-mêmes* (74%), *ne pas être à l’aise lorsqu’il s’agit de parler de soi* (73%) et *ne pas savoir comment obtenir de l’aide* (50%). Parmi les obstacles courants liés à la stigmatisation, mentionnons *la crainte de ce que la famille ou les amis pourraient penser.* Les étudiants ont indiqué qu’il est important de fournir du soutien au bien-être et des soins de santé mentale sur le campus, à des heures flexibles, et accessibles au moyen d’un système de réservation en ligne.

**Conclusions:**

la culture de la santé mentale adaptée aux étudiants peut constituer une approche durable pour éliminer les obstacles comportementaux et pratiques identifiés. Si ces obstacles sont réduits, on peut s’attendre à une augmentation de la demande de services et à la nécessité d’améliorer leur efficacité. Un énoncé de services clair, un point d’accès unique en ligne intégrant un système de triage pour orienter les étudiants vers les niveaux de soins indiqués et des protocoles de soins clairement définis, y compris vers les services communautaires, permettraient de mieux harmoniser le modèle de soins par paliers, d’améliorer l’efficacité et l’accès et de susciter des attentes réalistes en ce qui concerne le soutien à la santé mentale à l’université.

## Introduction

Over the past decade, there has been an increase in the reported prevalence of common mental health disorders in the general population of young people and in university students in Canada and other western countries.^1–4^ In parallel, universities have reported progressive increases in demand for mental health support, outpacing increases in enrolment,^5,6^ and straining resources.^5^ Universities acknowledge they have an important role to play in ensuring a welcoming learning environment that promotes student well-being, as well as providing accessible student-tailored well-being promotion and support for common mental health concerns.^7,8^ An important challenge for universities is to determine what scope of mental health support is feasible and appropriate to provide and how best to collaborate with community mental health for students who require more intensive specialized services and may not be connected to local health care.^8,9^

Authoritative papers and standards have recommended a combination of complementary whole-university and student-tailored services targeting common mild to moderate mental health concerns rationalized in accordance with a stepped care framework.^7,8,10,11^ There is recognition that the university should not attempt to duplicate community or specialized mental health services, but rather focus on developing, evaluating, and continually improving integrated, evidence-based well-being promotion and early intervention services.^4,6–8^

There is a paucity of Canadian research informing access to university mental health support, barriers to access, and support gaps from the student perspective. Yet, this information is essential to support development and continual improvement of resources and services.^8,12^ To address this need, we analyzed quantitative and qualitative data collected from successive cohorts of undergraduate students over their first year attending a large Canadian university to (i) describe the student experience of accessing university mental health services and (ii) summarize student perspectives of university mental health support barriers and gaps, and opportunities for improvement.

## Methods

### Data Source

This multiple cohort study utilized data from the U-Flourish Student Well-Being Survey, a digital biannual survey completed by first-year undergraduates at entry (mid-September) and completion (mid-March prior to final exams) of first year and repeated in subsequent years of study.^13,14^ All incoming first-year students were invited to complete a survey via their student email. Participation was encouraged through classroom announcements, in-person and online advertising, and booths at residences and campus-welcoming events. Coffee vouchers and the opportunity to win an iPad were offered as participation incentives.

The current analysis included qualitative and quantitative survey data from five cohorts of first-year students entering university from 2018 to 2022. Each cohort completed the year before, after, or during a unique stage of the COVID-19 pandemic that affected campus life. The first cohort (2018/2019) completed their first year entirely pre-pandemic, the second (2019/2020) switched to remote learning and support at the end of the academic year prior to final exams, the third (2020/2021) experienced the year completely remote during the peak of the pandemic, the fourth (2021/2022) experienced a hybrid of remote and in-person learning and service delivery, starting and finishing in person, and the fifth (2022/2023) completed the year entirely in post-pandemic conditions. The Fall baseline survey was completed by 25%–58% of students across cohorts (39% overall), and the Spring follow-up was completed by 33%–66% of those students (46% overall; Supplementary Figure 1). The average number of days between completing the surveys was 183 (SD = 7; range = 152–206).

The U-Flourish study was reviewed for ethical compliance by the Queen's University and Affiliated Teaching Hospitals Research Ethics Board (HSREB PSIY-692-20). Students provided informed consent to access the U-Flourish Survey.

### School Setting and Clinical Context

Queen's Student Wellness Services (SWS; https://www.queensu.ca/studentwellness/) comprise mental health and learning support services comparable to those found at most Canadian universities; namely Health Promotion, Disability, Counselling, and Health Services. This paper focuses on student access and perceived barriers and gaps related to mental health support provided by the University Counselling and Health Service.

### Survey Variables (Baseline Measures)

#### Demographic Characteristics

Students reported their age, self-identified gender^a^ (male, female, or gender diverse), and international student status. Program of study was obtained through deterministic linkage to the university administrative database.

#### Mental Health Problems

Personal and family history of mental illness was indicated from a list of diagnoses that included: mood, anxiety, psychotic, eating, neurodevelopmental, sleep and substance use disorder, attention-deficit/hyperactivity disorder, and learning difficulties. Lifetime history of self-harm (“hurt yourself on purpose without trying to end your life”) and suicidality (“thoughts about ending your life”, “made any suicide attempts”) were measured using items from the Columbia Suicide Rating Scale.^15^ Symptoms of depression in the prior 2 weeks were measured using the Patient Health Questionnaire (PHQ-9).^16^ Symptoms of anxiety were similarly measured using the Generalized Anxiety Disorder scale (GAD-7).^17^ On both the PHQ-9 and GAD-7 a score of 10 or more identified screen positives.^16,17^

### Survey Variables (Follow-up Measures)

#### University Services

Students reported whether they accessed university mental health services during the year. For each service accessed they were asked: (1) how easy was it to access (1 = very easy to 5 = very difficult), (2) how quickly were you seen following first contact (within 24 h, within a week, 1–2 weeks, 3–4 weeks, over a month), and (3) how helpful was it (1 = very helpful to 4 = not at all helpful).

#### Barriers to Care

Practical and attitudinal barriers to care were taken from the Barriers to Care Checklist,^18^ and completed by students who felt they needed help for their mental health but did not receive it, delayed reaching out, or did not continue receiving it during the year. Using the stigma subscale of the Barriers to Access to Care Evaluation Scale (BACE-3)^19^ students were asked if any of the items would (or have) stopped, delayed, or discouraged them from getting, or continuing with professional help for a mental health problem if they had experienced one (Yes, Most or All of the time vs. Not at all or Maybe/Sometimes).

#### Importance of Campus Mental Health Services

Students completing the 2022 spring survey (Cohort 4) ranked the importance (*Not* to *Very* or *Extremely Important*) of the university providing specific mental health services on campus, along with the importance of access to a counselling and primary care appointment through online booking.

#### Open-Text Survey Questions

Students who accessed mental health services were asked to provide short responses describing what they found particularly helpful or unhelpful about each service. In addition, all students were asked to provide brief, written responses to the following: (1) are there any campus mental health or wellness services currently not available that would be helpful and should be considered, (2) are there any currently offered services that were helpful and should be continued, and (3) should any currently offered services be changed or improved, and if so, why and which changes would you suggest?

### Analysis

Quantitative analyses were conducted using SAS Version 9.4 (SAS Institute, Cary, NC). Analyses were restricted to students who completed a baseline and spring survey and had data on whether they accessed university mental health services. The proportion of students with missing data on this item was 16% (792/4,930; Supplementary Figure 1). Students lost to follow-up or excluded due to missing data were slightly older on average (18.3 [SD = 2.0] vs. 18.2 [SD = 1.7], *t*-test *P* = .02) and more likely male identifying (34% vs. 25%, χ^2^ *P* < .01), but similar proportions screened positive for anxiety and/or depression at baseline (43.5% vs. 42.8%, χ^2^ *P* = .47). Person mean imputation was used if a single item was missing on the GAD-7 or PHQ-9. Descriptive analyses were initially conducted by cohort defined by year of entry to university. Given minimal differences identified between cohorts the combined analysis is presented, and any statistically significant differences (χ^2^ *P* < .05) are described in the text.

Student access to university mental health services was described by demographic characteristics and symptom levels, including the proportion accessing each service screening positive for anxiety or depression on the fall baseline survey. Student experiences with services and perceived barriers to care were similarly described.

Qualitative analysis was guided by interpretive description,^20^ and reflexive thematic analysis.^21^ Two qualitative researchers (KP and EC) initially read all open-text data and developed descriptive codes based on the first 100 participants per cohort. They then met with a third qualitative researcher (GD) to discuss and reflexively explore relationships between codes to develop a codebook. KP and EC utilized the codebook to complete coding. KP, EC, and GD met to generate first- and second-order themes by grouping coded data with similar meanings that were agreed upon by the full investigator team.

## Results

The average age of the 4,138 first-year student respondents at entry to university was 18.2 (SD = 1.7) years and the majority (74%) identified as female. A lifetime history of diagnosed mental illness was reported by 30%, and approximately one-third reported having a first-degree relative with a diagnosed mood, anxiety, psychotic, or substance use disorder. Taken together, 43% of students screened positive for clinically significant symptoms of anxiety (35%) and/or depression (32%), and over one-fifth (23%) reported a lifetime history of self-harm (Table 1).

**Table 1.** Description of the First-Year Undergraduate Student Sample, and the Proportions Accessing University Mental Health Services Over the Academic Year (Cohorts Spanning 2018 to 2023).

	Full sample		Students who accessed services		
	*n*	(col%)	*n*	(row%)	*P* ^a^
Overall	4138	(100)	620	(15.0)	
Age (at university entry)					<.001
≤17	815	(20.3)	114	(14.0)	.32
18	2709	(67.5)	386	(14.3)	.03
19	220	(5.5)	42	(19.1)	.10
≥20	268	(6.7)	65	(24.3)	<.001
Gender					<.001
Female	2960	(73.7)	498	(16.9)	<.001
Male	1028	(25.6)	86	(8.4)	<.001
Gender diverse	30	(0.8)	11	(36.7)	.003
International student					
Yes	299	(7.4)	42	(14.3)	.74
No	3730	(92.6)	564	(15.1)	
Program of study					<.001
Arts, Humanities & Social Sciences	1443	(35.4)	252	(17.5)	<.001
Life & Physical Sciences	1136	(27.9)	144	(12.7)	.01
Engineering & Applied Science	678	(16.6)	85	(12.5)	.05
Business	343	(8.4)	43	(12.5)	.21
Computing	148	(3.6)	10	(6.8)	.003
Professional Schools (Nursing, Medicine, Law)	326	(8.0)	78	(23.9)	.04
Lifetime history of mental illness ^b^					
Yes	1155	(30.1)	287	(24.9)	<.001
No	2685	(69.9)	305	(11.4)	
Family history of mental illness ^c^					
Yes	1382	(35.2)	264	(19.1)	<.001
No	2541	(64.8)	329	(13.0)	
Mental health symptoms at school entry					
Depression screen positive (PHQ-9 ≥ 10)					
Yes	1247	(31.9)	297	(23.9)	<.001
No	2668	(68.2)	291	(10.9)	
Anxiety screen positive (GAD-7 ≥ 10)					
Yes	1372	(35.0)	328	(24.0)	<.001
No	2551	(65.0)	263	(10.3)	
Lifetime history of self-harm					
Yes	909	(23.2)	222	(24.5)	<.001
No	3016	(76.8)	368	(12.2)	
Lifetime suicidal thoughts or behaviour					
Yes	1464	(37.3)	334	(22.9)	<.001
No	2461	(62.7)	256	(10.4)	

^a^ *P*-value from chi-square test comparing the proportion of students accessing services within each subgroup. ^b^ includes diagnosed mood, anxiety, psychotic, eating, neurodevelopmental, sleep, and substance use disorder, ADHD, and learning difficulties. ^c^ includes diagnosed mood, anxiety, psychotic, and substance use disorders.

### Quantitative Findings

#### Accessing University Mental Health Services

Of the students surveyed, 620 (15%) reported accessing services over first year. Access was lowest during the 2020–2021 academic year (10%) coinciding with the peak of the pandemic and highest the following 2 years (18% and 21%; Table 2). Older students and those identifying as gender diverse (37%) or female (17%) were more likely to access services than were younger or male students (8%; Table 1). Furthermore, students reporting a lifetime mental disorder and those who screened positive for anxiety, depression, self-harm, or suicidal thoughts and behaviours at entry were more likely to access services over first year. There was no difference in access by international status and patterns of access were similar across academic years.

**Table 2.** Campus Mental Health Services Accessed by First-Year Students, by Cohort/Academic Year and Stage of the Pandemic.

	Cohort 1 (2018/2019) Pre-pandemic		Cohort 2 (2019/2020) Transitional		Cohort 3 (2020/2021) Remote		Cohort 4 (2021/2022) Hybrid		Cohort 5 ^a^ (2022/2023) Post-pandemic	
Accessed campus mental health services	*n*	(%)	*n*	(%)	*n*	(%)	*n*	(%)	*n*	(%)
Yes	245	(13.7)	149	(15.3)	40	(10.0)	107	(17.8)	80	(21.0)
No	1789	(86.3)	824	(84.7)	360	(90.0)	495	(82.2)	301	(79.0)
Services accessed										
Program embedded counselling	33	(13.6)	58	(39.2)	7	(18.0)	26	(24.5)	38	(43.7)
Counselling at student wellness	171	(70.7)	89	(60.1)	28	(71.8)	59	(55.7)		
Nurse at student Health services	11	(4.6)	6	(4.1)	4	(10.3)	16	(15.1)		
Family physician at student health services	35	(14.5)	19	(12.8)	8	(20.5)	25	(23.6)	24	(27.6)
FP psychotherapist at student health services	7	(2.9)	3	(2.0)	4	(10.3)	5	(4.7)		
Psychiatrist at student health services	20	(8.3)	9	(6.1)	5	(12.8)	5	(4.7)	2	(2.3)
Student health services mental health team									20	(23.0)

^a^ (1) Program embedded and student wellness counselling combined on Cohort 5 survey (university counselling service), (2) Student health services mental health team includes nurse, FP psychotherapist, and social and crisis worker at student health services.

#### What University Mental Health Services Were Accessed?

The central University Counselling Service was most accessed (65% of the students who accessed support), followed by academic program Embedded Counselling (23%), and a family doctor (FP) at Student Health Services (16%). The proportion of students accessing support who screened positive for anxiety and/or depression ranged from 62% (embedded counsellor) to 79% (FP for a mental health concern; Supplementary Figure 2). Of the students who accessed services, 26% accessed multiple, of which 74% screened positive for clinically significant symptoms. The most common combination of services accessed was University Counselling and an FP at Student Health (31%).

#### Student Experience

Just over one-half of the students who accessed services reported they were easy to access (45%–69%), while about one-quarter reported they were difficult to access (11%–38%). Easy access was most often reported for the Embedded Counselling Service (69%) or seeing a nurse at Health Services (68%) and least often for accessing a psychiatrist (45%) through Health Services, which required a referral from an FP (Table 3). Difficult access was most often reported to see a psychiatrist and least reported to see an FP psychotherapist. Time to be seen varied by service (Table 3); the longest wait times were to see a psychiatrist or FP, with 58% and 38% of students reporting a time to be seen of 3 or more weeks, respectively. Around half of students reported the service accessed was helpful or very helpful (42%–63%), while under one-fifth indicated feeling the service was not at all helpful (11%–21%; Table 3).

**Table 3.** Description of Student Experiences with University Mental Health Services (Fall 2018 to Spring 2023).

	Counselling at SWS (*n* = 347)		Program embedded counselling (*n* = 124)		Nurse at SHS (*n* = 37)		FP at SHS (*n* = 111)		FP psychotherapist at SHS (*n* = 18)		Psychiatrist at SHS (*n* = 40)	
	%	(95% CI)	%	(95% CI)	%	(95% CI)	%	(95% CI)	%	(95% CI)	%	(95% CI)
How easy was it to access?												
Easy	58.8	(53.6–64.0)	68.6	(60.3–76.8)	67.6	(51.7–83.4)	57.7	(48.3–67.0)	61.1	(36.2–85.1)	45.0	(28.9–61.1)
Neither difficult nor easy	11.5	(8.2–14.9)	5.7	(1.5–9.8)	13.5	(2.0–25.1)	11.7	(5.6–17.8)	27.8	(4.9–50.7)	17.5	(5.2–29.8)
Difficult	29.7	(24.9–34.5)	25.8	(18.0–33.6)	18.9	(5.7–32.2)	30.6	(21.9–39.3)	11.1	(0.0–27.2)	37.5	(21.8–53.2)
How quickly were you seen?												
Within a week	51.9	(46.6–57.2)	62.9	(54.3–71.5)	73.0	(58.0–88.0)	40.5	(31.3–49.8)	50.0	(24.4–75.6)	22.5	(9.0–36.0)
1–2 weeks	17.3	(13.3–21.3)	18.6	(11.6–25.5)	13.5	(2.0–25.1)	21.6	(13.8–29.4)	16.7	(0.0–35.7)	20.0	(7.0–33.0)
3 weeks or more	30.8	(26.0–35.7)	18.6	(11.6–25.5)	13.5	(2.0–25.1)	37.8	(28.7–47.0)	33.3	(9.2–57.5)	57.5	(41.5–73.5)
Overall, how helpful was the resource?												
Very helpful/helpful	41.8	(36.6–47.0)	45.2	(36.3–54.0)	56.8	(40.0–73.5)	50.6	(39.9–61.3)	63.2	(39.3–87.0)	44.7	(28.2–61.3)
Somewhat helpful	40.1	(34.9–45.2)	36.3	(27.7–44.9)	29.7	(14.3–45.2)	34.5	(24.3–44.7)	26.3	(4.5–48.1)	34.2	(18.4–50.0)
Not at all helpful	18.2	(14.1–22.2)	18.6	(11.6–25.5)	13.5	(20–25.1)	14.9	(7.3–22.6)	10.5	(0.0–25.7)	21.1	(7.5–34.6)

*Note.* All values are percentages.

The following services were not directly measured on the Spring 2023 survey (Cohort 5): counselling at SWS, program embedded counselling, nurse at SHS, and FP psychotherapist at SHS.

SWS = student wellness services, SHS = student health services, FP = family physician.

#### Attitudinal Barriers to Accessing Care

**Figure 1.**
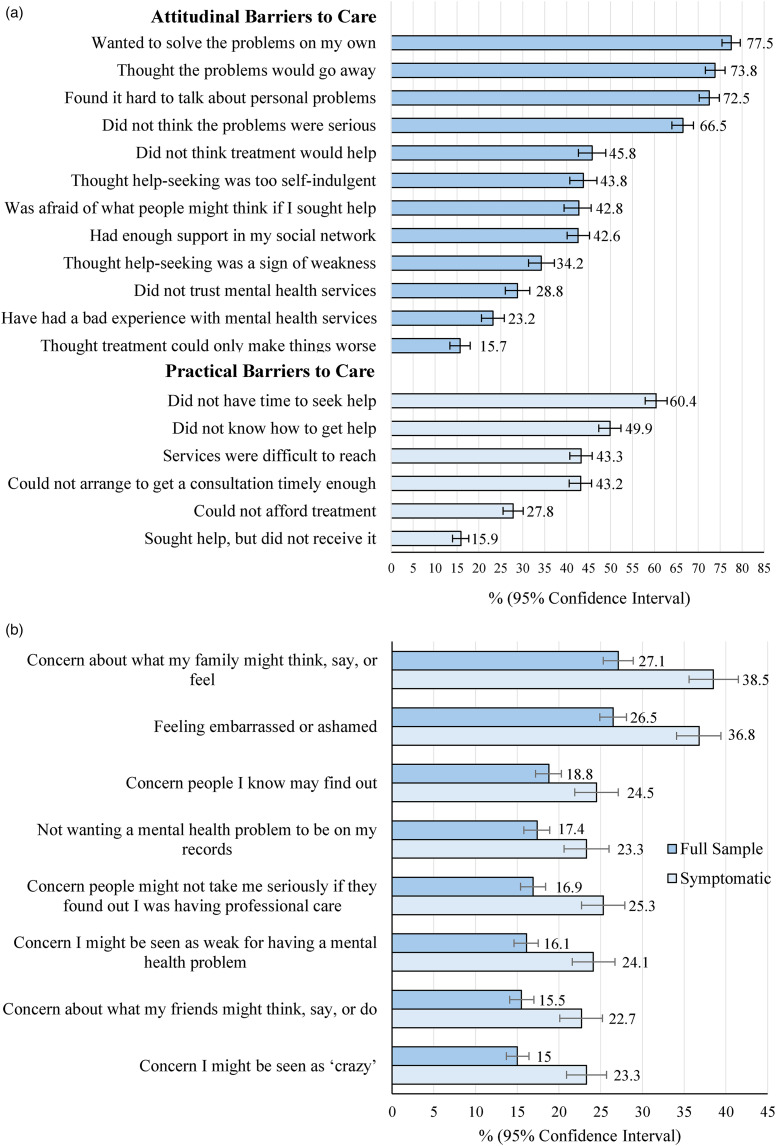
(a) the proportion of students who felt they needed help for their mental health or lifestyle but did not receive it, delayed reaching out, or did not continue to receive help during the academic year reporting attitudinal and practical barriers to care (*n* = 990–1,468). (b) The proportion of students reporting stigma barriers to accessing care “most” or “all of the time” (full sample and students who screened positive for anxiety and/or depression at school entry). Sample sizes ranged from 2,324 to 2,331 by barrier for the full sample, and from 1,026 to 1,029 for symptomatic students.

Commonly reported attitudinal barriers included “wanting to solve the problems on my own”, “thinking the problems would go away”, and “finding it hard to talk about personal problems” (Figure 1a). Findings were similar by gender identity, but a greater proportion of females than males reported having had a prior negative experience with services (25% vs. 16%, χ^2^ *P* = .02), and a greater proportion of males thought help-seeking was “too self-indulgent” (51% vs. 43%, χ^2^ *P* = .05). The proportion of students reporting they had enough support from their social network was lowest during the 2020–2021 peak pandemic year compared to other years (31% vs. over 40%, χ^2^ *P* ≤ .05).

#### Practical Barriers

Over half (60%; 95% CI: 58%–63%) of students who felt they needed help but did not receive it, delayed reaching out, or discontinued care endorsed not having time to seek help, and half (50%; 95% CI: 47%–52%) reported not knowing how to get help. Over a quarter (28%) felt they could not afford treatment, and 43% reported university services were difficult to reach, or that they could not arrange a timely enough appointment (Figure 1a). A greater proportion of females than males reported not having time to seek help (61% vs. 52%, χ^2^ *P* = .001), and that services were difficult to reach (45% vs. 33%, χ^2^ *P* = .001). The following barriers were more commonly reported after the onset of the pandemic (in 2020–2021, 2021–2022, and 2022–2023): “I did not know how to get help” (≥59% vs. ≤45%, χ^2^ *P* ≤ .001), and “I could not afford treatment” (≥34% vs. ≤25%, χ^2^ *P* ≤ .003). Although cost was a commonly reported barrier, mental health services offered by the university are of no direct cost to students.

#### Stigma as a Barrier to Care

The most common stigma barriers reported included “concerns about what my family might think, say, or feel” (27%), “feeling embarrassed or ashamed” (27%), and concern that “people I know may find out” (19%; Figure 1b). Compared to the full student sample, students screening positive for anxiety and/or depression were consistently more likely to report all stigma-based barriers. A greater proportion of females than males reported “concern about what my family might think, say or feel” (29% vs. 22%, χ^2^ *P* = .001), and “concern that I might be seen as crazy (16% vs. 12%, χ^2^ *P* = .02).

#### Services on Offer

Most students believed a range of services including counselling, psychotherapy, primary care (FP, nurses), and psychiatry were important to offer as part of university-based mental health services (Supplementary Figure 3). Less highly ranked but still important were peer-led supports. Of the 601 students surveyed, 88% reported that the ability to book online to access mental health services was “very important”.

### Qualitative Findings

#### Opportunities for Improving Services

**Figure 2.**
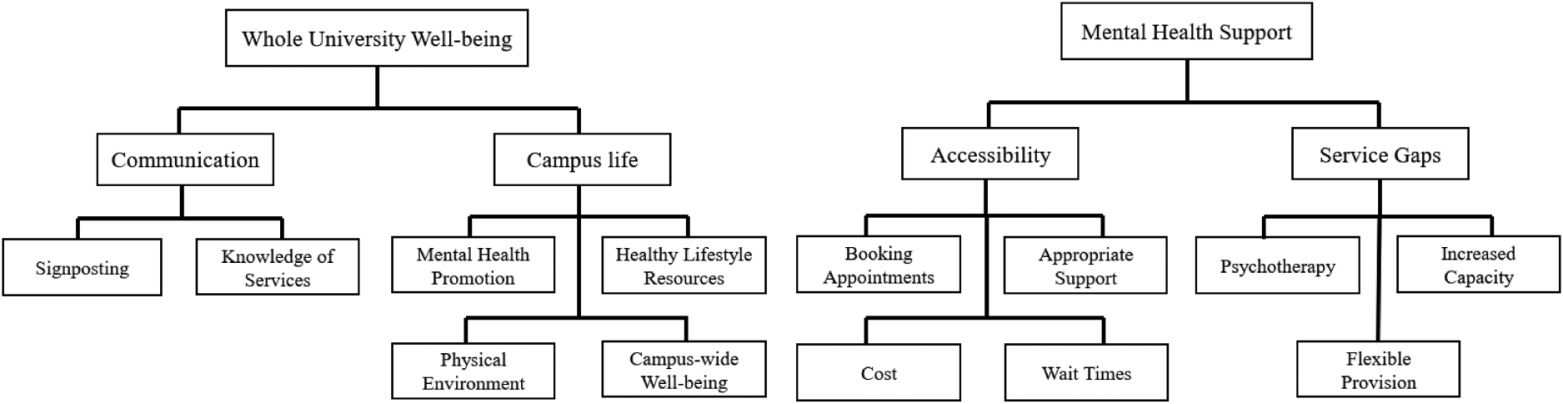
Student perceptions of opportunities for improving university mental health service.

Student open-text responses regarding improving mental health and well-being support aligned with two main themes: whole university well-being and mental health support (Figure 2). Within whole university well-being, subthemes identified included communication (i.e., a lack of awareness of available well-being resources and how to access them) and campus life (e.g., support for healthy lifestyle, pleasant physical environment, and mindful pedagogical practices). Within the mental health support theme, the two major subthemes identified were accessibility (i.e., accessing services, booking appointments, and being seen on a timely basis/long wait times) and gaps in services (i.e., need for longer-term support, increased capacity across services, access to psychotherapy in addition to short-term supportive counselling, and more flexible provision). A full description of the themes, subthemes, and categories along with supporting quotations are presented in Supplementary Table 1.

## Discussion

Over 40% of students screened positive for anxiety or depressive symptoms and a substantial proportion reported a lifetime mental disorder and/or self-harm/suicidal thoughts or behaviours at university entry, but only 15% accessed university mental health support over their first year. Commonly reported attitudinal and practical barriers to accessing services included a lack of understanding of common mental health symptoms and knowledge as to what services were available and how to access them. Moreover, despite significant efforts to reduce stigma at Canadian universities,^22^ over 35% of first-year students who screened positive for anxiety and/or depressive symptoms reported *concern about what their family might think, say, or feel* and being *too embarrassed or ashamed* to access support. Screen-positive status was high and comparable in students accessing different levels of university mental health support stratified by provider type. Furthermore, while one-half of students reported easy access to university services, almost 30% found access difficult. Students expressed the need for more timely access to a range of mental health services addressing a spectrum of mental health support needs with flexible hours and modes of delivery.

The findings in this study align with similar findings across universities in Canada and the UK in terms of high rates of common mental health concerns (clinically significant symptoms of anxiety and depression) reported among undergraduates,^2,4,23^ low rates of accessing support, and the existence of practical and attitudinal barriers to care.^4,23–25^ A somewhat surprising finding was the significant role that stigma continues to play in preventing or delaying students from accessing support when they think they would benefit from help.^22,26^

Students who screened positive for anxiety or depression were more likely to access services; however, screen-positive rates did not vary greatly across different levels of care proxied by provider type. This finding suggests there may be room for improving efficiency through rationalization of support access. Furthermore, among students accessing support, a higher proportion ranked accessing a family doctor or psychiatrist as “difficult”. This is not surprising given the higher intensity of care and corresponding reduced numbers of physicians and specialist physicians, but it may also indicate a misalignment between student expectation and understanding of their concern and clinically indicated level of support. While students with urgent and emerging severe mental illness require ready access to specialist care, symptoms in the mild to moderate range may benefit from short-term counselling, academic support, and/or self-guided online resources, rather than a course of more intensive individual psychological therapy and/or treatment with medication.^10^

Students indicated a desire to have a wide range of integrated mental health support services available on campus from well-being promotion to individualized mental health care. Student perceptions of well-being support highlight the need for universities to consider student well-being promotion as a campus-wide undertaking in co-partnership with faculty and students. That is our results align with extant literature, viewing student well-being and mental health as the product of students’ interactions with university systems, including academic, campus life, and student mental health and well-being services.^8^ In addition, students recognize the need for timely and accessible intervention of concerning mental health symptoms. Recommendations for a combination of coordinated whole university and stepped-care approaches in the provision of student mental health services support this sentiment.^6,7,10,11^ Campus mental health initiatives seem to be ever increasing but are often fragmented, not clearly justified or evaluated, and difficult to navigate.^7^ Rationalized access based on clinical need and appropriate for a university could improve efficiency and facilitate more timely access for those who would benefit the most from each service. Delay in accessing indicated support is associated with persistent and/or worsening mental health and academic outcomes,^7^ and should therefore be a priority in university mental health support development and planning.

This study had several strengths including the large broadly representative student sample covering different time periods prior to, during and after the pandemic. Barriers to care were collected using validated measures, and the inclusion of open-text questions added depth. Limitations of this study include attrition from baseline to follow-up, the predominance of female-identifying students (74% vs. approximately 60% of the student population),^27^ and the lower response rates observed during the peak of the pandemic. There is the potential for ascertainment bias, as youth with mental health concerns may have been more likely to respond. Quantitatively, only the experiences of students who successfully accessed services were captured, so difficulties accessing and wait times may be underestimated.

Implications of this research point to the potential importance of developing engaging and tailored student mental health literacy to reduce stigmatizing attitudes and improve knowledge and awareness about well-being and healthy coping, recognition of symptoms, and how, when, and where to reach out for support. Engaging and effective mental health literacy could facilitate students to make healthy lifestyle choices and for those experiencing mental health concerns to reach out sooner.^28^ Pilot findings suggest mental health literacy embedded in the curriculum and offered for credit is both of interest and benefit to students.^29^ In addition, repeated student-engaging information shared about campus mental health services throughout the academic year may help increase student awareness of available support options.

Furthermore, defining and publishing a justified and rationalized Statement of Student Wellness and Mental Health Services would provide students and their families, as well as mental health and healthcare providers, with an informed understanding and expectations to proactively plan for well-being and mental health support. For example, students with existing mental health concerns would then know what support was provided by the university and what community support they need to organize.

Finally, universities and students need to clearly recognize they cannot and should not provide all levels of mental health support. The formation of partnerships and care pathways between university and community mental health services seems paramount. Students are faced with major stresses, study in abbreviated academic terms, and are often away from established mental health support. Furthermore, community services, especially for transitional age youth, are often difficult to access.^7^

In summary, universities have an obligation to support student well-being and mental health, but there are limits to the level and nature of support that can and should be provided on campus. In terms of whole university wellness promotion efforts, these should be evidence-based, coordinated, and integrated into the very fabric of the institution from policy to pedagogical practices to social-cultural aspects of campus life.^8^ In addition to short-term problem-focused counselling, findings suggest that investment needs to be made in evidence-based individual and group psychological therapies and a multidisciplinary mental health team embedded in a university primary care practice.^7^ Furthermore, the student journey through different services could benefit from being more integrated (e.g., having shared electronic records), interactive, and collaborative (offered jointly by health promotion, disability, student health, and counselling services). Incorporating digital tools is one approach to facilitating information sharing across university services and providing a more joined-up and engaging student mental health support journey.^30^

In agreement with the National Standard,^12^ we posit that universities should focus on developing and providing effective, engaging, and tailored mental health promotion and university-based primary mental health support targeting early identification and intervention for common mental health concerns. It makes sense that prevention and early intervention are organized into a cohesive set of coordinated and integrated services starting with an accessible clinical triage process (online, in-person, and over the phone) that connects students to the appropriate level of care with therapeutic benefit from first contact. Such a triage process could help reduce time to be seen and fast-track students with more severe symptoms to the appropriate community mental health services, avoiding unnecessary delays associated with worsened prognosis and school failure.^7^ Triage alone, however, will not address the identified barriers and gaps in support. Services to support common mental health concerns need to be rationalized, integrated, regularly evaluated, and properly resourced.

## Supplemental Material

sj-docx-1-cpa-10.1177_07067437241295640 - Supplemental material for Access to University Mental Health Services: Understanding the Student Experience: L’accès aux services universitaires de santé mentale : comprendre l’expérience des étudiants Supplemental material, sj-docx-1-cpa-10.1177_07067437241295640 for Access to University Mental Health Services: Understanding the Student Experience: L’accès aux services universitaires de santé mentale : comprendre l’expérience des étudiants by Nathan King, William Pickett, Kurtis Pankow, Gina Dimitropoulos, Emma Cullen, Stephen McNevin, Scott B. Patten and Anne Duffy in The Canadian Journal of Psychiatry

sj-docx-2-cpa-10.1177_07067437241295640 - Supplemental material for Access to University Mental Health Services: Understanding the Student Experience: L’accès aux services universitaires de santé mentale : comprendre l’expérience des étudiants Supplemental material, sj-docx-2-cpa-10.1177_07067437241295640 for Access to University Mental Health Services: Understanding the Student Experience: L’accès aux services universitaires de santé mentale : comprendre l’expérience des étudiants by Nathan King, William Pickett, Kurtis Pankow, Gina Dimitropoulos, Emma Cullen, Stephen McNevin, Scott B. Patten and Anne Duffy in The Canadian Journal of Psychiatry

sj-docx-3-cpa-10.1177_07067437241295640 - Supplemental material for Access to University Mental Health Services: Understanding the Student Experience: L’accès aux services universitaires de santé mentale : comprendre l’expérience des étudiants Supplemental material, sj-docx-3-cpa-10.1177_07067437241295640 for Access to University Mental Health Services: Understanding the Student Experience: L’accès aux services universitaires de santé mentale : comprendre l’expérience des étudiants by Nathan King, William Pickett, Kurtis Pankow, Gina Dimitropoulos, Emma Cullen, Stephen McNevin, Scott B. Patten and Anne Duffy in The Canadian Journal of Psychiatry

sj-docx-4-cpa-10.1177_07067437241295640 - Supplemental material for Access to University Mental Health Services: Understanding the Student Experience: L’accès aux services universitaires de santé mentale : comprendre l’expérience des étudiants Supplemental material, sj-docx-4-cpa-10.1177_07067437241295640 for Access to University Mental Health Services: Understanding the Student Experience: L’accès aux services universitaires de santé mentale : comprendre l’expérience des étudiants by Nathan King, William Pickett, Kurtis Pankow, Gina Dimitropoulos, Emma Cullen, Stephen McNevin, Scott B. Patten and Anne Duffy in The Canadian Journal of Psychiatry

## Notes

a. The authors recognise that there is a distinction between sex and gender, in line with the Canadian Institutes of Health Research guidance (i.e., https://cihr-irsc.gc.ca/e/48642.html). The language used here and throughout the manuscript reflects language recommended by student collaborators, in which they recommended that when asking about gender identity the terms male and female be used.
