# Supplementary material for: Access to University Mental Health Services: Understanding the Student Experience: L’accès aux services universitaires de santé mentale : comprendre l’expérience des étudiants
Source: Can J Psychiatry. 2024 Nov 4;69(12):841–51. doi: 10.1177/07067437241295640 (PMC11562885; doi:10.1177/07067437241295640)
Supplement: sj-docx-1-cpa-10.1177_07067437241295640 - Supplemental material for Access to University Mental Health Services: Understanding the Student Experience: L’accès aux services universitaires de santé mentale : comprendre l’expérience des étudiants [file sj-docx-1-cpa-10.1177_07067437241295640.docx]

| INVITED TO PARTICIPATE IN U-FLOURISH FALL BASELINE SURVEY | |
| --- | --- |
| COHORT 1 (2018/2019): | 5,242 |
| COHORT 2 (2019/2020): | 5,123 |
| COHORT 3 (2020/2021): | 5,835 |
| COHORT 4 (2021/2022): | 5,787 |
| COHORT 5 (2022/2023): | 5,818 |
| **TOTAL:** | **27,805** |
|  | |
| RESPONDED TO FALL BASELINE SURVEY | |
| COHORT 1 (2018/2019): | 3,029 (58%) |
| COHORT 2 (2019/2020): | 2,949 (58%) |
| COHORT 3 (2020/2021): | 1,472 (25%) |
| COHORT 4 (2021/2022): | 1,991 (34%) |
| COHORT 5 (2022/2023): | 1,367 (24%) |
| **TOTAL:** | **10,808 (39%)** |
|  | |
| RESPONDED TO SPRING FOLLOW-UP SURVEY | |
| COHORT 1 (2018/2019): | 1,984 (66%) |
| COHORT 2 (2019/2020): | 1,165 (40%) |
| COHORT 3 (2020/2021): | 489 (33%) |
| COHORT 4 (2021/2022): | 819 (41%) |
| COHORT 5 (2022/2023): | 473 (35%) |
| **TOTAL:** | **4,930 (46%)** |
|  | |
| COMPLETED ITEM ON ACCESSING CAMPUS SERVICES (ANALYSIS SAMPLE) | |
| COHORT 1 (2018/2019): | 1,787 (90%) |
| COHORT 2 (2019/2020): | 971 (83%) |
| COHORT 3 (2020/2021): | 397 (81%) |
| COHORT 4 (2021/2022): | 602 (74%) |
| COHORT 5 (2022/2023): | 381 (81%) |
| **TOTAL:** | **4,138 (84%)** |

**Supplemental Figure 1**. Participation Flow Chart
