# Supplementary material for: Access to University Mental Health Services: Understanding the Student Experience: L’accès aux services universitaires de santé mentale : comprendre l’expérience des étudiants
Source: Can J Psychiatry. 2024 Nov 4;69(12):841–51. doi: 10.1177/07067437241295640 (PMC11562885; doi:10.1177/07067437241295640)
Supplement: sj-docx-2-cpa-10.1177_07067437241295640 - Supplemental material for Access to University Mental Health Services: Understanding the Student Experience: L’accès aux services universitaires de santé mentale : comprendre l’expérience des étudiants [file sj-docx-2-cpa-10.1177_07067437241295640.docx]

**Supplementary Figure 2.** The proportion (and number) of first year students who screened positive for anxiety or depression at entry to university and accessed mental health support broken down by service (Fall 2018 to Spring 2022)
