# Supplementary material for: Access to University Mental Health Services: Understanding the Student Experience: L’accès aux services universitaires de santé mentale : comprendre l’expérience des étudiants
Source: Can J Psychiatry. 2024 Nov 4;69(12):841–51. doi: 10.1177/07067437241295640 (PMC11562885; doi:10.1177/07067437241295640)
Supplement: sj-docx-3-cpa-10.1177_07067437241295640 - Supplemental material for Access to University Mental Health Services: Understanding the Student Experience: L’accès aux services universitaires de santé mentale : comprendre l’expérience des étudiants [file sj-docx-3-cpa-10.1177_07067437241295640.docx]

**Supplemental Figure 3**. Student rankings of the importance of having access to the mental health service on campus (n= 598 to 601). Responses are from first-year students who completed the Spring 2022 Survey (Cohort 4).
