# Supplementary material for: Access to University Mental Health Services: Understanding the Student Experience: L’accès aux services universitaires de santé mentale : comprendre l’expérience des étudiants
Source: Can J Psychiatry. 2024 Nov 4;69(12):841–51. doi: 10.1177/07067437241295640 (PMC11562885; doi:10.1177/07067437241295640)
Supplement: sj-docx-4-cpa-10.1177_07067437241295640 - Supplemental material for Access to University Mental Health Services: Understanding the Student Experience: L’accès aux services universitaires de santé mentale : comprendre l’expérience des étudiants [file sj-docx-4-cpa-10.1177_07067437241295640.docx]

| **Supplemental Table 1.** Description of the qualitative findings on students’ perceptions of opportunities for improving mental health services | | | |
| --- | --- | --- | --- |
| **Theme** (description) | **Sub-Theme** (description) | **Category**  (description) | **Representative Quote(s)** (participant ID) |
| **Whole University Well-being**  This theme reflects student recommendations that allude to a need for a ‘whole-campus’ approach to student mental health | **Communication**  Students wanted to be more aware of services available on campus related to mental health | **Signposting**  Students wanted the university to put more effort into advertising available services to students. | “I have no idea what is provided, due to lack of marketing of them [i.e., mental health services]” (p1590)  “More advertising for mental health support. I hear nothing about it and have no idea where to get help” (p1597) |
|  |  | **Knowledge of services**  Students asked for services to be added that were already available suggesting they are unaware of many services. Quotes here include examples of students requesting services that are already offered. | “I don't know if this is offered, but LGBTQ+ counsellors that specifically help queer students on any of their problems (anxiety, depression, etc. not just being queer)” (p988)  “Family doctors available for students who need one who can do referrals and do check-ups” (p1424) |
|  | **Campus life**  Students expressed that a variety of non-clinical interventions related to campus life would be beneficial to their mental health | **Mental health promotion**  Students wanted non-clinical opportunities to maintain their mental health like discussions with non-clinicians, self-guided digital resources, therapy animals, art programs, and social groups. | “Maybe a safe space to vent your thoughts while someone listens/sympathizes with you where necessary” (p1511)  “There should be consistent dog/pet therapy” (p1621)  “ArtHive - in person activities. More times offered to engage in art to destress” (p1632)  “A wellness group where students can talk about daily stresses and support each other through discussions about coping skills, etc.” (p1660) |
|  |  | **Healthy lifestyle resources**  Students wanted healthy lifestyle options to be more accessible, such as more nutritious cafeteria food, easier access to physical activity opportunities, and access to other healthy lifestyle resources. | “I think exercise programs should be available, free, drop-in” (p1463)  “Healthier food options can play a huge role on mental health, cafeteria food should be improved to match needs with people who feel reliant on a healthy lifestyle, as well as having nutritionist advise healthy options” (p1241) |
|  |  | **Physical environment**  Students wanted the built environment at the university to incorporate designs that promote mental health such as better lighting and more greenspace. | “I am not sure if they are available but SAD [seasonal affective disorder] lamps for rent during the winter (or available at the library for use when studying) would be great” (p1575)  “I think a dedicated green space on campus (preferably indoor given one school term being over the winter months) would be an amazing place for students to go relax, study, and complete assignments” (p1699) |
|  |  | **Campus-wide Well-being**  Students wanted more compatibility between services on campus. Students felt that there was a disconnect between student wellness programs and academic programs, which led to challenges for their mental health. They expressed that compatibility across departments and services would help their mental health, as well as more mental health awareness from academic staff. | “Being sent back and forth between departments was not something I could handle emotionally and as a result my grades suffered immensely. Making the temporary accommodations and support process very clear from the start (aka having a direct email or line) would take the stress off of people’s shoulders” (p1546)  “Better mental health support education for profs” (p1635)  “Mental health check ins from professors” (p1747) |
| **Mental Health Support**  Students thought mental health support services could be improved through improving accessibility and adding new or improving existing mental health services. | **Accessibility**  Students noted current services were often difficult to access due to issues with booking, capacity, cost, or wait times. | **Booking Appointments**  The booking process (phone only) and times possible to book (standard business hours) were a source of frustration for students. | “Scheduling appointments shouldn’t only be over the phone, students should be able to book appointments online or even in-person, because a lot of people have phone anxiety and/or can’t call when the office is open” (p1721)  “I think that the appointments should become an online booking system where you can see the times and book it accordingly, takes the stress off reception and eliminates lengthy phone waits” (p1669) |
|  |  | **Appropriate Support**  Students felt it was difficult to get an appointment with a care provider about their mental health concerns. | “Embedded counselling is… very overbooked. At least in [faculty] it is almost impossible to book an appointment” (p1588)  “The length of the meetings and the fact that they were in the middle of the afternoon was inconvenient for my schedule. Shorter bi-weekly evening meetings would have been better for me specifically” (p1674)  “More integrated counseling within faculties because it is helpful but there is a very long wait” (p1726) |
|  |  | **Cost**  Students felt the cost of services were prohibitive to accessing appropriate services. | “$500/year for psychotherapy is woefully insufficient. As someone who has been receiving therapy on and off for over a decade, and who continues to live with well-managed mental illnesses, I attribute my success to my access to therapy” (p1574) |
|  |  | **Wait times**  When students were able to get an appointment with a care provider, they felt the wait was often too long. | “It is so hard to get an appointment. Sometimes I have to wait for a month” (p1421)  “The wait times to get therapy appointments discourage me from consulting help when needed” (p1731) |
|  | **Service Gaps**  Students requested modifications to current service offerings or new services. | **Flexible service provision**  Students wanted the university to offer their own text message help services and online appointments. | “Maybe some sort of online counselling service through queens, because for me personally I would have maybe sought some help online rather than in person as its more private, less intimidating, and takes up less valuable time from studying, homework, etc...” (p232)  “Private texting chat (immediate that you can access straight from your phone number)” (p1591) |
|  |  | **Psychotherapy**  Students wanted to be able to access long term psychotherapy through student wellness. | “Long term counselling options, as opposed to short term, crisis management type counselling” (p1099) |
|  |  | **Increased clinician capacity**  Students wanted more available clinicians for on-campus services. | “I have chosen to use other services in Kingston. Why is there only one doctor working [on campus] at a time? Hire at least 2.” (p413) |
